# Chromosome-Level Assembly Reveals a Fifteen-Chromosome Aneuploid Genome and Environmental Adaptation Strategy of Chinese Traditional Medical Fungus *Wolfiporia hoelen*

**DOI:** 10.3390/ijms25168786

**Published:** 2024-08-13

**Authors:** Shoujian Li, Bing Li, Shunxing Guo

**Affiliations:** The Institute of Medicinal Plant Development, Chinese Academy of Medical Sciences & Peking Union Medical College, Beijing 100193, China; 13756298450@163.com (S.L.); zudengtianxia@126.com (B.L.)

**Keywords:** edible and medicinal mushroom, aneuploid genome, chromosome number, adaptive evolution, meiosis

## Abstract

The sclerotia of *Wolfiporia hoelen* are one of the most important traditional Chinese medicines and foods commonly used in China, Japan, Korea, and other Asian countries. To provide a high-quality reference genome and deepen our understanding of the genome of *W. hoelen* to elucidate various biological phenomena. In this study, we assembled three genomes of *W. hoelen* using a combination of Nanopore and Illumina sequencing strategies. The fifteen-chromosome genome L7 of *W. hoelen* was assembled with two-sided telomere and rDNA sequences for the first time. The chromosome count was subsequently confirmed through collinearity analysis, correcting the previous belief that *W. hoelen* had only fourteen chromosomes. Moreover, the aneuploid genome was discovered in *W. hoelen* for the first time through sequencing depth analysis of different chromosomes, and only some strains of *W. hoelen* exhibit aneuploid genomes. According to the genome analysis of homokaryotic offspring and protoplast-isolated strains, a potential variation in chromosome allocation patterns was revealed. Moreover, the gene function enrichment analysis of genes on reduplicated chromosomes demonstrated that aneuploidy in the genome may be the result of environmental adaptation for *W. hoelen*. The discovery of an aneuploid genome also provides new ideas for genetic improvement of *W. hoelen*.

## 1. Introduction

Genomics is the core and foundation of life science research and the pioneering direction of future scientific and technological innovation. With the advancement of sequencing and assembly technologies, numerous genomes of edible and medicinal macrofungi have been assembled and published. Not only widely cultivated mushrooms like *Lentinula edodes* [1], *Auricularia heimuer* [2], *Agaricus bisporus* [3], and others [4,5], but also some newly domesticated species such as *Oudemansiella raphanipes* [6] and *Naematelia aurantialba* [7] have had their genomes published. Meanwhile, the genome assembly level has risen significantly. Some species have been assembled to the chromosome or near-chromosome level [8,9], and some have been assembled to the T2T level [10]. Meanwhile, the chromosome number of more and more species has been revealed [11]. Unlike plants and animals, the chromosome number in fungi is difficult to observe due to their small size [12]. For the study of chromosome numbers, genetic linkage maps, macroscopic observations, pulsed-field gel electrophoresis, and high-throughput chromosome conformation capture can all be used [13,14,15,16,17,18].

Eukaryotes organize their genomes into chromosomes, with each organism having its own specific karyotype, originally defined by the number and appearance of the chromosomes in the nucleus [19]. Euploidy refers to a chromosome number that is an integral multiple of the chromosome number for a haploid cell [20]. Aneuploidy is a deviation from a balanced genome caused by either the gain or loss of chromosomes, leading to an unbalanced genomic state [21]. The aneuploid phenomenon was discovered in plants, animals, and also fungi [22,23,24]. The yeast fungi *Saccharomyces cerevisiae*, *Candida albicans*, and *Cryptococcus neoformans* have all been demonstrated to have widely aneuploid genomes [25,26,27], which can be artificially induced under adverse environmental conditions [28,29,30]. Recently, the aneuploid phenomenon was discovered in the dimorphic edible mushroom *Tremella fuciformis*, which includes yeast and mycelial forms. This phenomenon was demonstrated to be related to the scarce formation of basidiospores [31].

*Wofiporia hoelen* (Fr.) Y.C. Dai and V. Papp [32,33] is a traditional Chinese medicine known as ‘Fuling’ in China. It grows underground on the roots of pines and is distributed in East Asia [34]. The sclerotia of *W. hoelen* are edible and medicinal tissues widely used as traditional crude drugs in China, South Korea, and Japan. They can also be consumed as food [35,36]. With the advancement of pharmacy, various pharmacological activities of the asexual sclerotia of *W. hoelen* have been demonstrated, including diuresis, tranquilization [37], antioxidant effects [38], antitumor properties [39], anti-inflammatory effects [40], and immunomodulatory effects [41].

As an important and unique species capable of forming sclerotia. To further reveal the evolution and formation mechanism of sclerotia, and mechanisms of function metabolite synthesis, its genome was published in 2020 for the first time [42]. Subsequently, Cao et al. [43], Kim et al. [44], and Zhang et al. [45] successively published the genome of *W. hoelen*. Meanwhile, we also assembled and published the homokaryotic genome SS20 of *W. hoelen* based on the establishment of homokaryotic strain isolation and identification methods [46,47]. Strain SS20 is a homokaryotic strain, and it was also the first homozygous genome assembled in our previous study [8]. In addition, Cao et al. first proposed the 14 chromosomes of *W. hoelen* based on microscopic observations [43]. And we further agreed with the results based on the telomere numbers and Hi-C-assisted assembly. This is the first chromosome-level genome of *W. hoelen*. Even though the basic information of the *W. hoelen* genome has been revealed, the relation between genome and biological phenomenon is still little known, such as the different strains have distinct homokaryotic offspring types [48], and hybrid strains often demonstrate poorer growth performance compared to the parent strains.

In the present study, we assembled three different genomes of *W. hoelen* using a combination of Nanopore long-read sequencing and Illumina short-read sequencing strategies to achieve chromosomal-level genomes. The complete chromosomal-level genome of *W. hoelen* with 15 chromosomes was assembled here. This study corrected previous findings that *W. hoelen* has 14 chromosomes. The aneuploid genomes of *W. hoelen* were discovered, and the aneuploid phenomenon of various *W. hoelen* strains and homokaryotic offspring was analyzed. This study revealed a potential variation in chromosome allocation patterns. Gene function analysis of genes on reduplicated chromosomes revealed the possible reasons for the formation of aneuploid genomes in *W. hoelen*. This study will deepen the understanding of genomics, genetics, and the evolution of macrofungi, and provide new insights for the genetic improvement of *W. hoelen*.

## 2. Results and Discussion

### 2.1. Chromosome-Level Genome Assembly of Wolfiporia hoelen Strain L7

**Figure 1:**
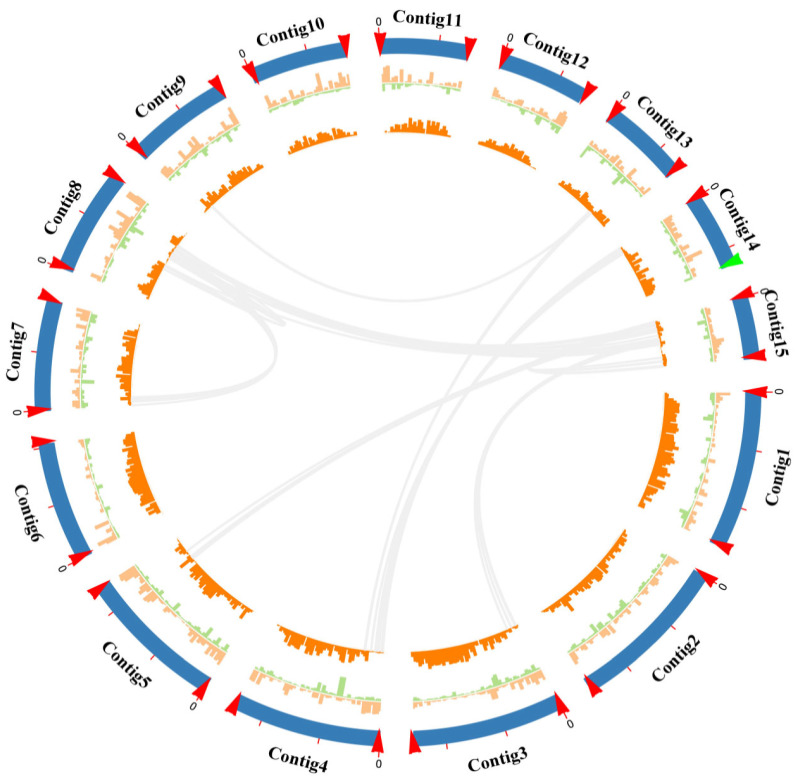
Circos graph of genome characteristics of *Wolfiporia hoelen*. From the outer ring to the inner ring are 15 chromosomes, transposable elements, retrotransposons, gene density, and large fragment duplication. The red arrow points to telomere sequences, and the green arrow points to rDNA sequences.

**Figure 2:**
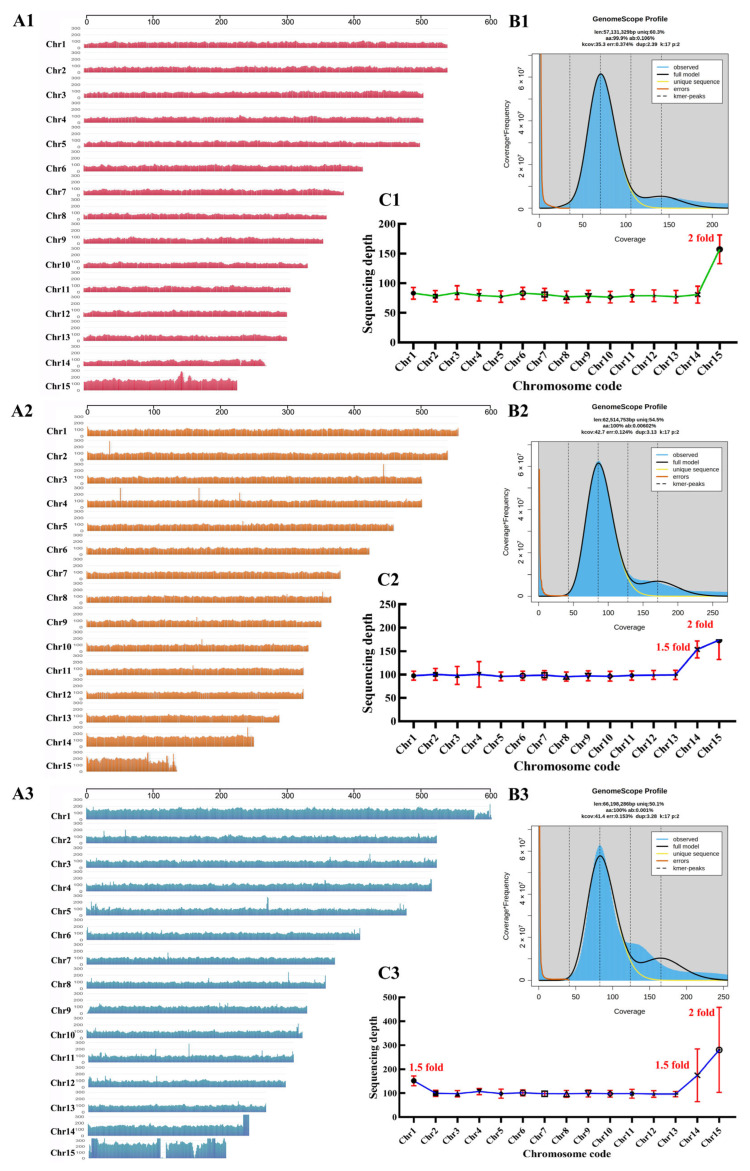
Sequencing depth for 15 chromosomes based on nanopore sequencing reads and k-mer analysis of different genomes based on Illumina sequencing reads of *Wolfiporia hoelen*. Sequencing depth of genome L7 (**A1**), Pr2C (**A2**), and PrT (**A3**). k-mer analysis results of genome L7 (**B1**), Pr2C (**B2**), and PrT (**B3**). Line charts of sequencing depth of different chromosomes for genome L7 (**C1**), Pr2C (**C2**), and PrT (**C3**).

To further assemble a high-quality genome of *W. hoelen* based on a previous study [8], the homokaryotic strain L7 was utilized for genome sequencing. The sequencing was performed using the Illumina NovaSeq 6000 platform and the PromethION platform. Homokaryotic strain L7 was isolated from strain L12, which was derived from a distinct strain with a previously published SS20 genome [8]. A de novo genome assembly was performed by integrating Nanopore long-read sequencing and Illumina short-read sequencing (Figure 1). The predicted genome size is 57.13 Mb. There was no apparent heterozygous peak, and the heterozygosity was low at 0.106% based on the k-mer (k = 17) analysis (Figure 2(B1)).

A genome of 59.68 Mb was assembled based on 294,029 Nanopore reads (~85×, 5.08 Gb data size), which consists of 28 contigs with a contig N50 ~4.11 Mb, with the longest contig being 5.46 Mb. The mapping rate of Illumina data is 99.83%, the genome coverage is 99.94%, and 97.9% of complete BUSCOs all indicate the high genome integrity of L7. Furthermore, the telomere and rDNA analysis showed 14 contigs of genome L7 containing double-sided telomeres, and 1 contig containing single-sided telomeres and rDNA, indicating that the genome L7 has been assembled to the chromosome level (Figure 1). In total, 96.98% of sequences were deemed as chromosomes (57.88 Mb), with the longest chromosome 5.46 Mb and the shortest 2.15 Mb.

### 2.2. Genome Comparison and Collinearity Analysis of Different Wolfioria hoelen Strains

Meanwhile, the genomes of Pr2C and PrT were also sequenced and assembled, which were all homokaryotic strains and have low genome heterozygosity (Figure 2(B2,B3)). For strain Pr2C, a genome of 58.40 Mb was assembled based on 2,422,147 Nanopore reads (~136×, 7.94 Gb data size), which consists of 27 contigs with a contig N50 ~4.23 Mb, with the longest contig being 5.57 Mb. The mapping rate of Illumina data is 94.36%, the genome coverage is 99.90%, and 97.9% of complete BUSCOs all indicate high genome integrity. For strain PrT, a genome of 57.64 Mb was assembled based on 638,818 Nanopore reads (~126×, 7.26 Gb data size) and consists of 17 contigs with a contig N50 ~4.11 Mb, with the longest contig being 6.05 Mb. The mapping rate of Illumina data is 89.04%, the genome coverage is 99.93%, and 97.9% of complete BUSCOs indicate high genome integrity (Table 1).

When comparing the three genomes, it is important to consider factors such as genome size, contig N50, percentage of repeat sequences, genome coverage, and the presence of complete BUSCOs. These parameters provide insights into the accuracy of genome assembly. The mapping ratio of Illumina data for L7 is higher than that of Pr2C and PrT, indicating the high quality of the L7 genome. Furthermore, 13 contigs with double-sided telomeres, 1 contig with single-sided telomere and single-sided rDNA, and 2 contigs with single-sided telomeres were discovered in the Pr2C genome, while 11 contigs with double-sided telomeres and 5 contigs with single-sided telomeres were identified in the PrT genome (Table S1). In addition, the distribution of sequencing depth in genome L7 showed high consistency, but in the genomes Pr2C and PrT, the sudden fluctuations in sequencing depth were sporadic (Figure 2). This also indicates the high-quality assembly of genome L7.

Compared to the SS20 genome we published previously as the highest quality genome [8], the number of contigs has significantly decreased from 78 to 27/28 contigs. The contig N50 was significantly extended from 3760 kb to over 4100 kb (Table 1). The complete chromosomes contain telomeres on both ends. Genome L7 (15 chromosomes) has more chromosomes than genome Pr2C (13 chromosomes), which has more chromosomes than genome PrT (11 chromosomes), and more chromosomes than genome SS20 (9 chromosomes) (Table S1). This demonstrates the higher quality of the newly assembled genomes compared to genome SS20. Meanwhile, the longest chromosome of the SS20 genome, anchored according to Hi-C data, was 7.47 Mb [8], significantly larger than the largest chromosome found here. This suggests that chromosome 1 of SS20 may have been incorrectly assembled.

**Figure 3:**
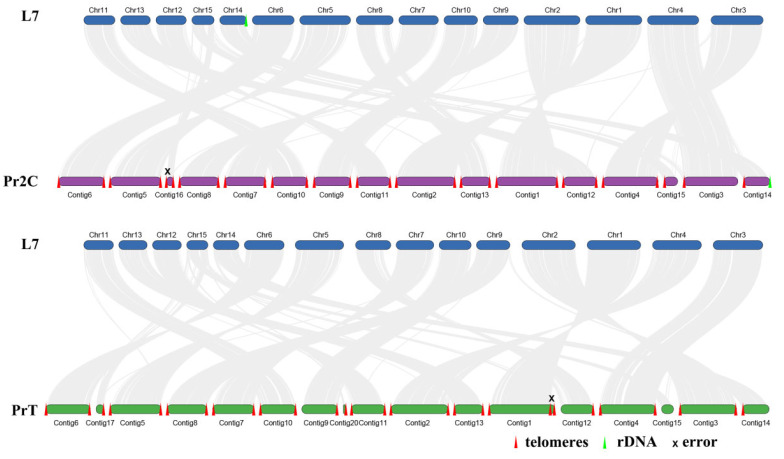
Whole-genome protein collinearity of *W. hoelen* strain L7, PrT, and Pr2C.

Moreover, the collinearity analysis was conducted for the three genomes based on protein identity (Figure 3). The majority of chromosomes in L7 exhibit high collinearity with the contigs of Pr2C and PrT. In total, 13 contigs of the Pr2C genome exhibit high collinearity with the L7 genome, and 10 contigs of the PrT genome show high collinearity with the L7 genome. This indicates that the corresponding contigs have been assembled at the chromosome level. Meanwhile, some contigs exhibit high collinearity with genome L7 but lack a single-sided telomere, such as contig3 of genome Pr2C, and contig9 and contig12 of genome PrT. The most challenging chromosome to assemble is Chr15, as the corresponding contigs of the Pr2C and PrT genomes were scattered. Contig15, contig17, and contig 20 of the PrT genome, as well as contig 16 and contig 15 of the Pr2C genome, exhibit high collinearity with Chr15 in the L7 genome. The collinearity analysis revealed no breaks in the chromosomes of genome L7. This suggests that *W. hoelen* has 15 chromosomes, which were exclusively assembled using the OLC (Overlap-Layout-Consensus) algorithm. While there are some assembly mistakes, contig1 of the PrT genome has three telomeres, and contig16 of Pr2C is significantly shorter than the corresponding Chr15, and has double-sided telomeres. The 15 chromosomes of *W. hoelen* revealed here correct the previous results of 14 chromosomes [8].

### 2.3. Aneuploidy Genome of Wolfiporia hoelen

Based on determining the number of chromosomes, the ploidy of the *W. hoelen* genome was further studied. Sequencing depth of reads can reflect the phenomenon of aneuploid genomes. The sequencing depth of genomes L7, Pr2C, and PrT was analyzed based on Nanopore sequencing reads (Figure 2). The obvious aneuploidy phenomenon was discovered in *W. hoelen*. Chromosome 15 of genome L7 has 2× (fold) sequencing depth compared to other chromosomes. Chromosomes 14 and 15 of genome Pr2C have 1.5× and 2× sequencing depth, respectively, compared to other chromosomes. Chromosome 1 of genome PrT has an additional 1.5× sequencing depth compared to other chromosomes based on genome Pr2C. This is the first discovery of genomic aneuploidy in *W. hoelen*.

Further, the sequencing depth of genomes L7, Pr2C, and PrT was analyzed based on Illumina sequencing reads. When comparing the sequencing depth analyzed with Nanopore reads, the sequencing depth analyzed with Illumina reads exhibited less consistency, with more abrupt fluctuations between high and low sequencing depth regions. While the Illumina sequencing depth can also reflect the aneuploidy phenomenon and yield similar results (Figure S1).

Due to the strain L7 being isolated with a single spore, the aneuploid genome of L7 may have been caused by abnormal chromosome allocation during meiosis or by the parent strain L12 also having an aneuploid genome. To strains Pr2C and PrT, which were isolated with protoplasts, the aneuploidy phenomenon reflects that the original strain L14 also had an aneuploid genome. Therefore, some parental strains were used to conduct aneuploidy genome analysis.

### 2.4. Aneuploidy Characters for Different-Type Heterokaryotic Strains

To further reveal the aneuploidy characteristics in species *W. hoelen*, the heterokaryotic strains isolated from cultivated or wild sclerotia were sequenced. The sequencing depth of different chromosomes was analyzed, and genome L7 was used as the reference genome.

**Figure 4:**
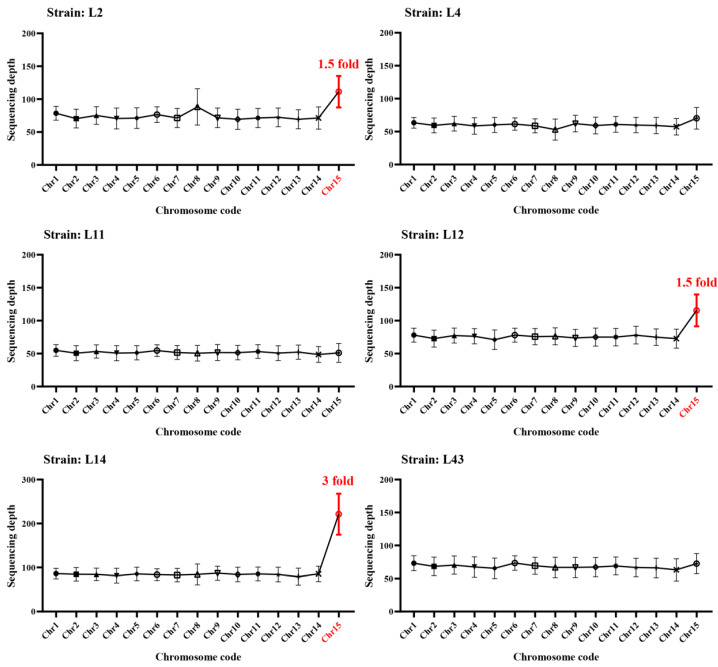
Sequencing depth of different chromosomes for different heterokaryotic strains mapping to genome L7. The red chromosome code presents the reduplicated chromosome, and the red number indicates the reduplicated folds.

In the six sequenced strains, three strains (L2, L12, L14) exhibit an aneuploidy phenomenon, where the duplicated chromosomes are all from chromosome 15. The other three strains have euploid genomes (Figure 4). Chromosome 15 of genomes L2 and L12 has 1.5× sequencing depth compared to other chromosomes, while chromosome 15 of genome L14 has 3× sequencing depth compared to other chromosomes (Figure 4). In the species *W. hoelen*, some strains have euploid genomes, while others have aneuploid genomes.

In our previous results, the strains can be divided into two types, each with a different homokaryotic strain type [49]. For Type I strains, the homokaryotic strain was significantly different from its parent strain, showing a slower growth rate and less aerial mycelia. The homokaryotic strain of Type II strains shows no significant difference from its parent strain. It grows rapidly and has thick aerial mycelia. Among the six strains used in this study, L2, L4, and L14 were type I strains, while L11, L12, and L43 were type II strains. Notably, L2, L12, and L14 exhibited aneuploid genomes, whereas L4, L11, and L43 exhibited euploid genomes (Figure 4). This indicates that the strain types are not directly related to euploid or aneuploid genomes.

### 2.5. Aneuploidy Characters for Homokaryotic Offspring of L12 and L14

To determine if chromosome allocation is normal during meiosis, the genomes of homokaryotic offspring were sequenced. The sequencing depth of different chromosomes was analyzed. For homokaryotic offspring of strain L12, the genome L7 was used as the reference genome, and the genome Pr2C was used as the reference genome for homokaryotic offspring of strain L14.

**Figure 5:**
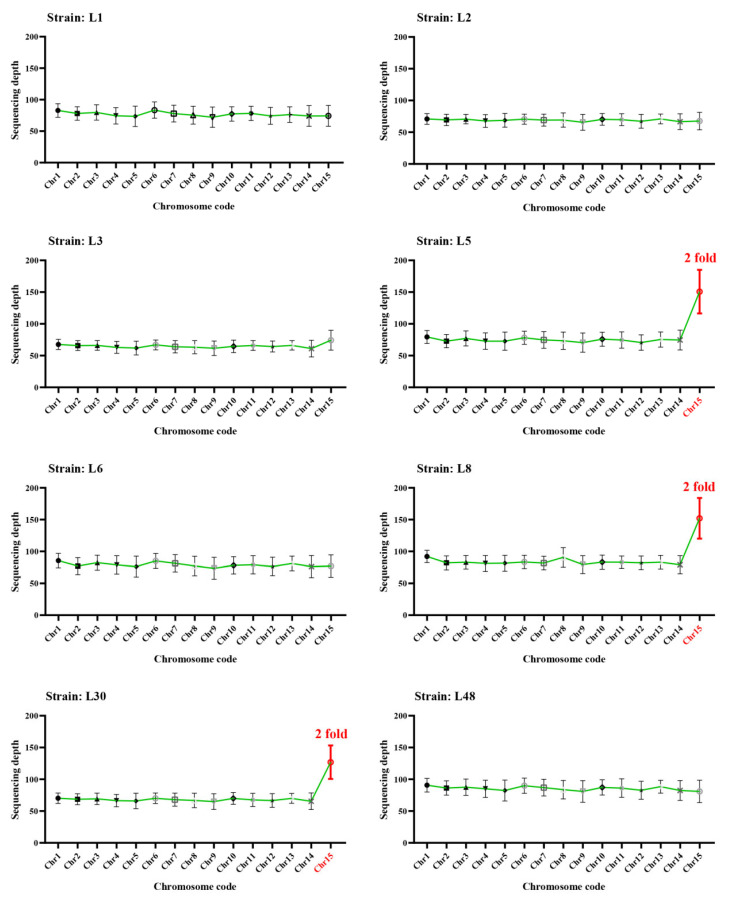
Sequencing depth of different chromosomes for homokaryotic offspring of L12. The red chromosome code presents the reduplicated chromosome, and the red number indicates the reduplicated folds.

**Figure 6:**
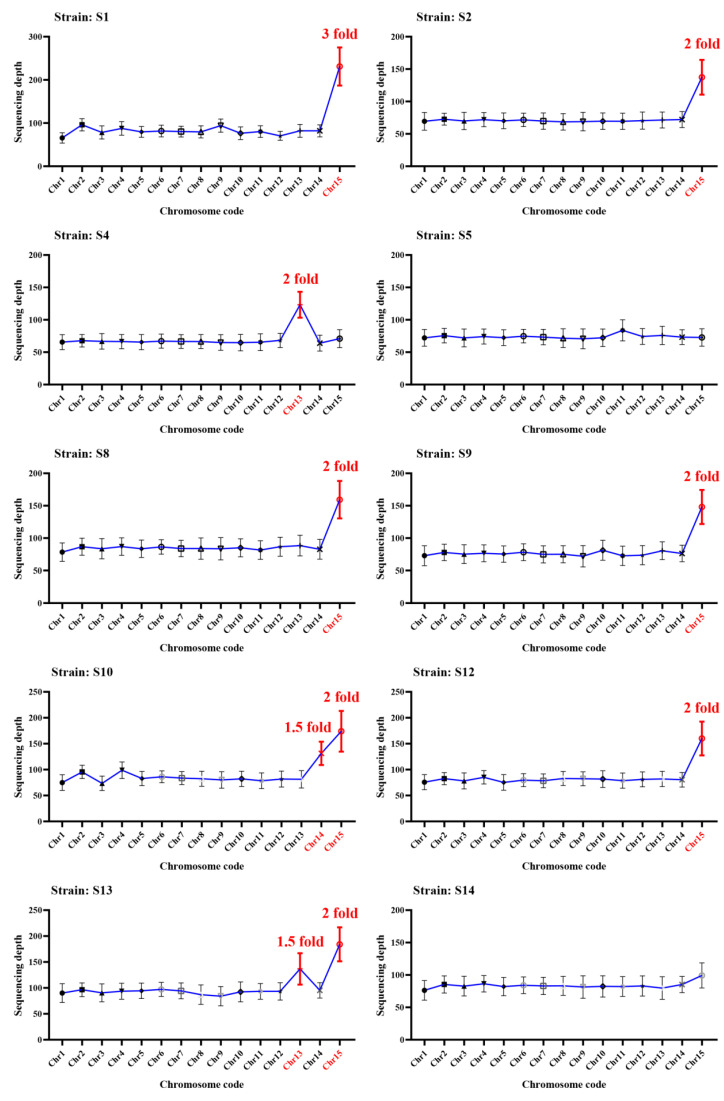

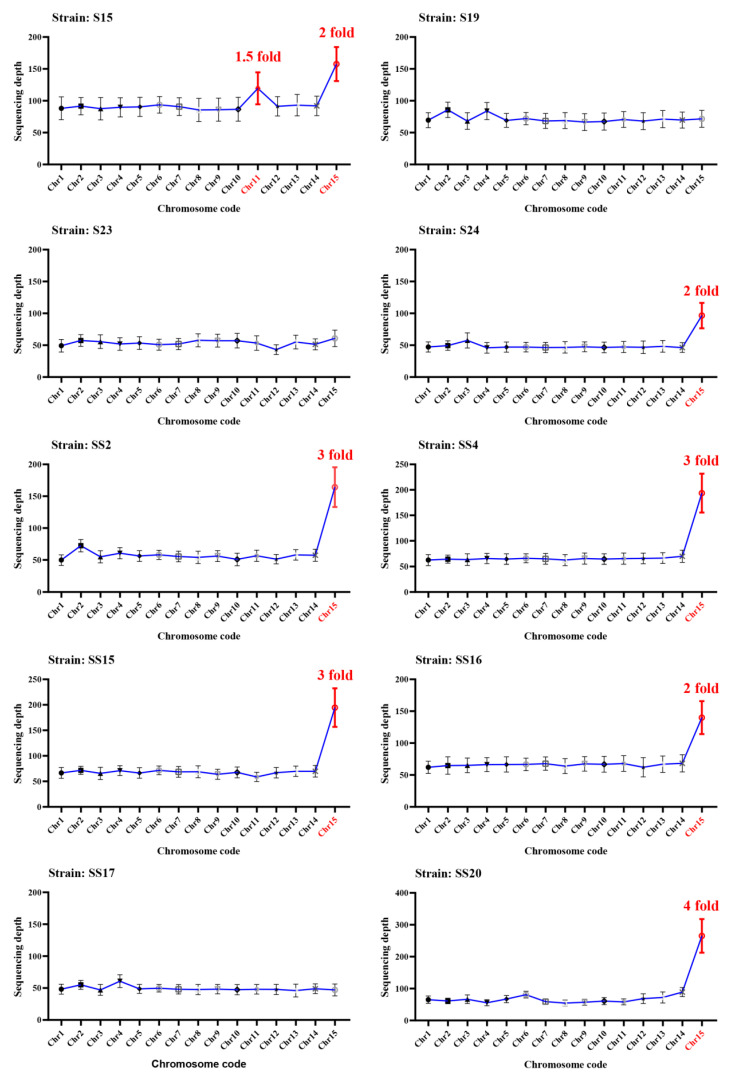
Sequencing depth of different chromosomes for homokaryotic offspring of L14. The red chromosome code presents the reduplicated chromosome, and the red number indicates the reduplicated folds.

Eight homokaryotic offspring of strain L12 were further sequenced and analyzed. Among them, three of which have 2× sequencing depth for chromosome 15 compared to other chromosomes, while the other five strains exhibited a euploid genome with similar sequencing depth across all chromosomes (Figure 5). Meanwhile, twenty homokaryotic offspring of strain L14 were further sequenced and analyzed. Among them, fifteen strains exhibited aneuploid genomes, while five strains showed euploid genomes. Among the fifteen offspring strains with aneuploid genomes, some strains only exhibit one chromosome duplication. Specifically, ten strains have 2× sequencing depth for chromosome 15 compared to other chromosomes, four strains have 3× sequencing depth for chromosome 15 compared to other chromosomes, one strain has 4× sequencing depth for chromosome 15 compared to other chromosomes, and one strain has 2× sequencing depth for chromosome 13 compared to other chromosomes. Moreover, some strains have chromosome duplication for more than one chromosome. For strain S10, chromosome 14 has a sequencing depth of 1.5×, and chromosome 15 has a sequencing depth of 2×. For strain S13, chromosome 13 has a sequencing depth of 1.5×, and chromosome 15 has a sequencing depth of 2×. Lastly, for strain S15, chromosome 11 has a sequencing depth of 1.5×, and chromosome 15 has a sequencing depth of 2× (Figure 6).

According to the law of normal distribution of chromosomes. If the genome of L12 contains two types of nuclei, one containing a euploid genome (15 chromosomes), and the other containing an aneuploid genome (15 chromosomes + Chr15). The homokaryotic offspring would only include strains with a euploid genome (15 chromosomes) or with an aneuploid genome (15 chromosomes + Chr15). This was in accordance with the results of genome analysis for nine homokaryotic offspring of strain L12, among which five have euploid genomes, and four have aneuploid genomes (including strain L7). Whereas strain L14 exhibited reduplication only on chromosome 15, the homokaryotic offspring showed reduplication on chromosomes 11, 13, and 14 except chromosome 15. That presents the strain L14 may have a disordered chromosome distribution. That may be related to different types of *W. hoelen* strains [48]. In general, if a chromosome were to be reduplicated, the corresponding nucleus would lack a chromosome, leading to death due to chromosome loss. That may be the reason why strains with absent chromosomes were not obtained.

However, for the strains Pr2C and PrT, isolated with protoplasts, chromosome 14 was reduplicated, and in PrT, chromosome 1 was also reduplicated, except for chromosome 15 (Figure 2(A2,A3)). The homokaryotic strains isolated with protoplasts may have more than one nucleus with the same mating type. The results showed that chromosomes 1 and 14 may also be reduplicated in some nuclei. Even though the L14 strain only exhibited reduplication of chromosome 15, the various nuclei in strain L15 may possess different genome types, resulting in diverse genomes with varying chromosome reduplication patterns. To reveal the actual chromosome distribution law and genome variations among different nuclei of the same strain, conducting additional genome analyses on strains isolated with protoplasts may provide the answers in follow-up studies.

### 2.6. Gene Function Enrichment of Genes on Chromosome 15

In the aneuploid genome, the gene dosage effects were prominent [21]. In the isolated strains from wild or cultivated sclerotia, only chromosome 15 was duplicated, albeit with varying multiples. Gene function enrichment was conducted for genes located on chromosome 15. On the whole, 54 genes are enriched in the ‘biological process’, 120 genes are enriched in the ‘molecular function’, and 107 genes are enriched in the ‘cellular components’. For biological processes, the majority of genes are enriched in metabolic process (16), cellular process (15), and single-organism process (10). For molecular function, the majority of genes are enriched in catalytic activity (62) and binding (52). For cellular components, the majority of genes are enriched in the cell (19), membrane (17), and organelle (13) (Figure S2).

**Figure 7:**
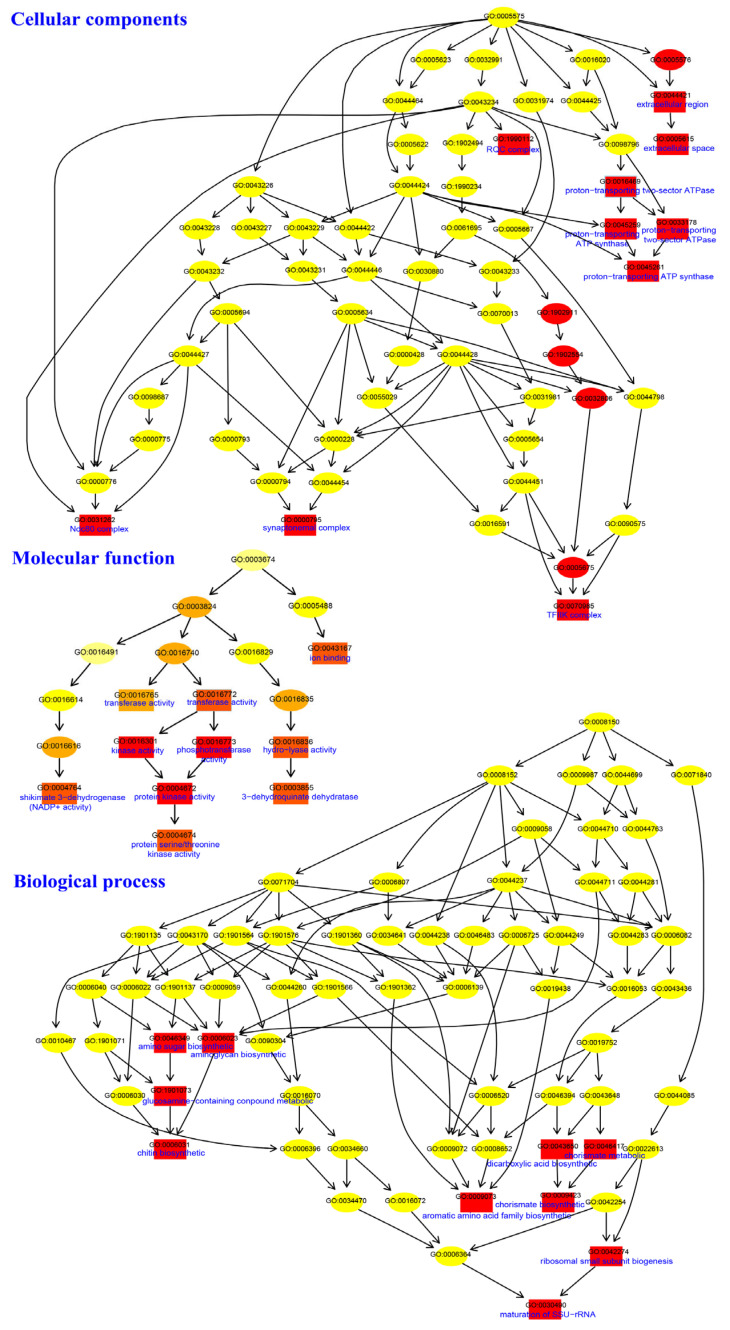
GO enrichment analysis of genes on chromosome 15. The rectangle represents enriched first ten GO items, and the ellipse represents other items. Red represents obvious enriched, followed by orange and yellow.

Specifically, according to the directed acyclic graph (DAG), the key gene functions are associated with the ‘GO:0031262 Ndc80 complex’, ‘GO:0000795 synaptonemal complex’, ‘GO:0070985 TFIIK complex’, ‘GO:1990112 RQC complex’, ‘GO:0005615 extracellular space’, and ‘GO:0045261 proton-transporting ATP synthase complex’ for cellular components. ‘GO:0003855 3-dehydroquinate dehydratase activity’, ‘GO:0004764 shikimate 3-dehydrogenase (NADP+) activity’, ‘GO:0043167 ion binding’, and ‘GO:0004674 protein serine/threonine kinase activity’ for molecular function. ‘GO:0006031 chitin biosynthetic process’, ‘GO:0030490 maturation of SSU-rRNA’, ‘GO:0009073 aromatic amino acid family biosynthetic process’, and ‘GO:0009423 chorismate biosynthetic process’ for biological process (Figure 7).

According to the results, the enriched Gene Ontology (GO) terms of genes on chromosome 15 are mainly associated with environmental stress responses, such as salt stress, high or low-temperature stress, drought stress, heavy metal stress, and pathogen infection (Table 2). Moreover, chitin being one of the cell wall components, chitin biosynthesis was also enriched (Figure 7). As the cell wall is closely related to the outer environment, chitin biosynthesis must play an important role in resisting adverse environments. The study demonstrated that chromosome reduplication in *W. hoelen* primarily contributes to environmental adaptation. On the one hand, *W. hoelen* is widely distributed or grown from the northeast to the south of China, and it can adapt to various environments. On the other hand, the sclerotia grow underground, coming into contact and interacting with numerous other microorganisms. The chromosome reduplication and aneuploid genome formation of *W. hoelen* may indicate an adaptation to the environments.

In our previous results, chromosome 14 of the SS20 genome was found to play an important role in adapting to harsh environments. The genome features a high ratio of transposons, low gene density, low GC content, absence of single-copy homologous genes, and a collection of resistance genes [8]. Because Chr15 was considered to have similar functions, the gene density, repetitive sequence ratio, and GC content were analyzed. It was found that chromosome 15 has a high ratio of repetitive sequences, low gene density, low GC content, and a concentration of resistance genes (Table S2). The reduplicated chromosome 15 was identified as the homologous chromosome of chromosome 14 in the SS20 genome, which was classified as an accessory chromosome in the species *W. hoelen* [8]. This further demonstrated the relationship between chromosome 15 and environmental adaptation.

## 3. Materials and Methods

### 3.1. Homokaryotic Strain Isolation

The strains CGMCC 5.545 (L14), CGMCC 5.55 (L12), and NBRC 30268 (L11) were kindly provided by Hubei Provincial Hospital of Traditional Chinese Medicine. Strains CGMCC 5.137 (L2), L12, and L14 were preserved at the General Microbiological Culture Collection Center (CGMCC). Strain L4 was isolated from sclerotia cultivated in Yunnan Province, while strain L43 was isolated from wild sclerotia collected from Hubei Province. The homokaryotic strain was isolated using our previous methods [47,85] and confirmed through specific polymorphic loci of the *rpb2* gene [47]. Homokaryotic strains Pr2C and PrT were isolated using protoplasts from strain CGMCC 5.545, while other homokaryotic strains were obtained through single-spore isolation. All the strains were maintained on potato dextrose agar medium (PDA: 200 g/L potato, 20 g/L glucose, 18 g/L agar) at 4 °C for future experiments.

### 3.2. Genome Sequencing, Assembly, and Quality Assessment

The mycelia sample was prepared using our previous methods [8]. The total genomic DNA was extracted using the conventional cetyltrimethylammonium bromide method, and its quality was assessed using the Nanopore One spectrophotometer (NanoDrop Technology, Wilmington, DE, USA) and the Qubit 3.0 Fluorometer (Life Technologies, Carlsbad, CA, USA). For the Illumina and Oxford Nanopore library preparation, the VAHTS^®^ Universal Plus DNA Library Prep Kit for Illumina v2 and the SQK-LSK110, EXP-NBD104/114 kits were used separately. The library preparation followed standard protocols for each kit. The Illumina NovaSeq 6000 platform and PromethION platforms (Oxford Nanopore Technologies, Oxford, UK) were used for genome sequencing at Wuhan Benagen Tech Solutions Company Limited (Benagen, Wuhan, China).

Raw sequencing data generated by the PromethION platform underwent quality assessment and were processed using Oxford Nanopore GUPPY (version 0.3.0) to eliminate unsuccessful reads. The remaining passed reads were used for further analysis. For genome assembly, the software NECAT (version v0.0.1) (https://github.com/xiaochuanle/NECAT) (accessed on 10 August 2024) was used for preliminary genome assembly, and Racon (version 1.4.11) was utilized for error correction based on ONT sequencing data for two rounds. Further, the software Pilon (version 1.23) was used for error correction based on Illumina sequencing data for two rounds [86]. The Circos graph of the genome was generated using the ‘Advanced Circos’ module of TBtools v 2.086 [87]. The heterozygosity of the genome was estimated based on analysis of k-mer depth distribution using Jellyfish5 [88] and GenomeScope6 [89] for Illumina data. The integrity of chromosome-level genome assembly was estimated by BUSCO [90].

### 3.3. Telomere and rDNA Sequences Location

Telomere sequences of (CCCCTAA)n or (TTAGGGG)n [91] were analyzed, and the chromosome number was predicted based on the telomere count. To identify the ribosomal DNA (rDNA) regions within the genome, 28S rRNA (GenBank ID: NG_042623.1), 5.8S rRNA (GenBank ID: NR_111007.1), and 18S rRNA (GenBank ID: NG_063315.1) from *Saccharomyces cerevisiae* were used as a BLAST query [92].

### 3.4. Annotation of Gene Structure and Function

The combination of ab initio prediction, homology-based prediction, and transcriptome-assisted prediction was used to identify the genes encoding proteins. The software Exonerate (version 2.4.0) was used for homology-based prediction, Augustus (version 3.3.2), Genescan (version 1.0), and GlimmerHMM (version 3.0.4) were used for ab initio prediction, while StringTie (version 2.1.4) and TransDecoder (version 5.1.0) were utilized for transcriptome-assisted prediction. Finally, the software MAKER (version 2.31.10) was used to integrate gene prediction results.

For gene functional annotation, 9 databases were used, including UniProt (https://www.uniprot.org), Pfam (http://pfam.xfam.org/), RefSeq (https://www.ncbi.nlm.nih.gov/refseq/) (accessed on 20 August 2023), Non-Redundant Database (NR, https://ftp.ncbi.nlm.nih.gov) (accessed on 20 August 2023), InterProScan (https://github.com/ebi-pf-team/interproscan) (accessed on 20 August 2023), Clusters of Orthologous Genes (COG, http://www.ncbi.nlm.nih.gov/COG/) (accessed on 20 August 2023), Kyoto Encyclopedia of Genes and Genomes (KEGG, http://www.genome.jp/kegg/) (accessed on 20 August 2023), and Pathway and Gene Ontology (GO, http://geneontology.org) (accessed on 20 August 2023), Kyoto Encyclopedia of Genes and Genomes (KEGG, http://www.genome.jp/kegg/) (accessed on 20 August 2023) databases.

### 3.5. Whole-Genome Collinearity Analysis

To explore the number of chromosomes, whole-genome collinearity was conducted among the three genomes of *W. hoelen* sequenced and assembled in this study: Pr2C, PrT, and L7. The ‘One Step MCScanX’ and ‘Multiple Synteny Plot’ commands in the ‘Comparative Genomics module’ of the TBtools software (v 2.086) were used for the analysis and visualization of collinearity results [87].

### 3.6. Genome Resequencing

The sample preparation methods are the same as those in our previous study [46]. A total of 34 strains were subjected to genome resequencing, including 6 heterokaryotic strains isolated from sclerotia, 20 homokaryotic offspring isolated from strain L14, and 8 homokaryotic offspring isolated from strain L12. The genome was sequenced using the high-throughput sequencing instrument DNBSEQ-T7 (BGI, Shenzhen, China).

### 3.7. Sequencing Depth Analysis

The filtered clean data were mapped to the assembled genome using Minimap2 v2.1 [93] for data generated by Nanopore sequencing, while BWA 0.7.17 [94] was utilized for Illumina and DNBSEQ-T7 data. Samtools 0.1.19 was used to transfer BAM files, sort, and index them [95]. Furthermore, the sequencing depth was calculated using Sambamba 0.6.6 for all 10 kb bins of the genome [96]. The sequencing depth data were processed and graphed using WPS Excel version 17147 (WPS Office, Beijing, China) and GraphPad Prism 8.0.2 (Boston, MA, USA).

## 4. Conclusions

In conclusion, we have successfully assembled a complete aneuploid chromosome-level reference genome of *W. hoelen* for the first time and revealed that the formation of aneuploid genomes may be related to environmental adaptation. Firstly, the first chromosomal-level genome, L7, was assembled here using only the Overlap-Layout-Consensus algorithm. This study revealed 15 chromosomes with double-sided telomere and rDNA sequences for the first time, correcting the previous findings that *W. hoelen* has 14 chromosomes. Secondly, the aneuploid genomes of *W. hoelen* were discovered for the first time, and the ploidy of homokaryotic offspring and different *W. hoelen* strains were studied. The study revealed aneuploidy phenomena in different *W. hoelen* strains and discovered potential variations in chromosome allocation during the meiosis process of *W. hoelen*. Thirdly, the gene enrichment analysis was conducted for the genes on reduplicated chromosomes, revealing that the formation of aneuploid genomes of *W. hoelen* may be associated with environmental adaptation.

## Figures and Tables

**Table 1:** Genome characteristics of strains of *W. hoelen*.

	L7	Pr2C	PrT	SS20
Sequencing strategy	Illumina NovaSeq 6000 and Oxford Nanopore	Illumina NovaSeq 6000 and Oxford Nanopore	Illumina NovaSeq 6000 and Oxford Nanopore	Illumina Novaseq 6000 and PacBio
Genome size (Mb)	59.68	58.40	57.64	64.44
Number of Contigs	28	27	17	78
Number of Chromosomes	15 (OLC)	-	-	14 (HI-C)
N50 of Contigs (kb)	4114.4	4228.1	4105.74	3760
Max Contig (kb)	5459.1	5572.1	6050.4	5.29 Mb
Anchored to chromosome (Mb)	57.88	-	-	58.26
Number of protein-coding genes	10,421	11,085	10,749	10,567
Average gene length (bp)	2907.02	2416.16	2605.85	2004
Percentage of repeat sequences (%)	44.2	43.47	43.68	
Mapping rate of Illumina data (%)	99.83	94.36	89.04	
Genome coverage (%)	99.94	99.90	99.93	
GC content (%)	52.07	52.03	52.04	50.15
Complete BUSCO (%)	97.9	97.9	97.9	97.36
Reference	This study	This study	This study	[8]

**Table 2:** GO enrichment of genes on chromosome 15 and related response process.

GO Term and Related Response Biological Process	Reference
**GO: 0003855 3-dehydroquinate dehydratase activity**	
tea tree response to *Didymella bellidis* infection	[50]
*Reaumuria soongorica* response to low and high salt stress	[51]
*Fagopyrum tataricum* response to lead stress	[52]
*Paeonia lactiflora* response to drought stress	[53]
*Pseudomonas fluorescens* response to *Fusarium graminearum* infection	[54]
**GO: 0004674 protein serinethreonine kinase activity**	
maize response to drought stress	[55]
maize response to cadmium stress	[56]
*Forsythia suspensa* response to *Alternaria alternata* infection	[57]
foxtail millet response to *Sclerospora graminicola* infection	[58]
*Prunus dulcis* response to low-temperature stress	[59]
oat response to cold stress	[60]
rice response to *Megnaporthe oryzae* infection	[61]
mulberry response to *Colletotrichum* infection	[62]
*Pterocarya stenoptera* response to drought stress	[63]
**GO: 0004764 shikimate 3-dehydrogenase(NADP+) activity**	
tea tree response to *Didymella bellidis* infection	[50]
*Reaumuria soongorica* response to low and high salt stress	[51]
*Fagopyrum tataricum* response to lead stress	[52]
*Paeonia lactiflora* response to drought stress	[53]
*Broussonetia paperifera* response to cadmium stress	[64]
**GO: 0009423 chorismate biosynthetic process**	
*Triticum aestivum* response to drought and high-temperature stress	[65]
*Coinithyrium minitans* response to *Scelrotinia* infection	[66]
*Manihot esculenta* response to drought stress	[67]
*Pseudomonas fluorescens* response to *Fusarium graminearum* infection	[54]
**GO: 0031262 Ndc80 complex**	
*Malus domestica* response to drought and salt stress	[68]
**GO: 0009073 aromatic amino acid family biosynthetic process**	
maize response to cadmium stress	[56]
mulberry response to *Colletotrichum* infection	[62]
*Broussonetia paperifera* response to cadmium stress	[64]
*Triticum aestivum* response to drought and high-temperature stress	[65]
*Coinithyrium minitans* response to *Scelrotinia* infection	[66]
*Brassica napus* resist response to *Sclerotinia sclerotiorum* infection	[69]
maize response to biotic and abiotic stress	[70]
rice response to cold stress	[71]
potato response to *Phytophthora infestans* infection	[72]
apple response to *Diplocarpon mali* infection	[73]
*Musa* response to *Fusarium oxysporum* f.sp. *cubense*	[74]
**GO: 0043167 ion binding**	
maize response to drought stress	[55]
maize response to cadmium stress	[56]
foxtail millet response to *Sclerospora graminicola* infection	[58]
mulberry response to *Colletotrichum* infection	[62]
*Medicago sativa* response to low-temperature stress	[75]
*Populus cathayana* response to salt stress	[76]
*Echeveria* response to low-temperature stress	[77]
*Solanum lycopersicum* response to salt stress	[78]
*Sansevieria trifasciata* var. *laurentii* response to benzene stress	[79]
**GO: 0043650 dicarboxylic acid biosynthetic process**	
mulberry response to *Colletotrichum* infection	[62]
cucumber response to salt stress	[80]
rice response to high nighttime temperature stress	[81]
*Solanum tuberosum* response to *Fusarium sulphureum* infection	[82]
**GO: 0045261 proton-transporting ATP synthase**	
tea tree response to *Didymella bellidis* infection	[50]
*Brassica napus* resist response to *Sclerotinia sclerotiorum* infection	[69]
sweet sorghum response to soda stress	[83]
rice response to high-temperature stress	[84]

## Data Availability

The genomes of L7, Pr2C, and PrT were deposited in NCBI with the accession numbers: JBDKWP000000000, JBDKWQ000000000, and JBDKWR000000000.

## Supplementary Materials

The following supporting information can be downloaded at: https://www.mdpi.com/article/10.3390/ijms25168786/s1.

## References

[1] Yu H.L., Zhang L.J., Shang X.D., Peng B., Li Y., Xiao S.J., Tan Q., Fu Y.P. (2022). Chromosomal genome and population genetic analyses to reveal genetic architecture, breeding history and genes related to cadmium accumulation in *Lentinula edodes*. BMC Genom..

[2] Yuan Y., Wu F., Si J., Zhao Y.F., Dai Y.C. (2019). Whole genome sequence of *Auricularia heimuer* (Basidiomycota, Fungi), the third most important cultivated mushroom worldwide. Genomics.

[3] Sonnenberg A.S.M., Gao W., Lavrijssen B., Hendrickx P., Sedaghat-Tellgerd N., Foulongne-Oriol M., Kong W., Schijlen E.G.W.M., Baars J.J.P., Visser R.G.F. (2016). A detailed analysis of the recombination landscape of the button mushroom *Agaricus bisporus* var. bisporus. Fungal Genet. Biol..

[4] Qu J.B., Zhao M.R., Hsiang T., Feng X.X., Zhang J.X., Huang C.Y. (2016). Identification and characterization of small noncoding RNAs in genome sequences of the edible fungus *Pleurotus ostreatus*. BioMed Res. Int..

[5] Li H., Shi L., Tang W.Q., Xia W.W., Zhong Y.L., Xu X.Y., Xie B.G., Tao Y.X. (2022). Comprehensive genetic analysis of monokaryon and dikaryon populations provides insight into cross-breeding of *Flammulina filiformis*. Front. Microbiol..

[6] Zhu L.P., Gao X., Zhang M.H., Hu C.H., Yang W.J., Guo L.Z., Yang S., Yu H.L., Yu H. (2023). Whole genome sequence of an edible mushroom *Oudemansiella raphanipes* (Changgengu). J. Fungi.

[7] Sun T., Zhang Y.X., Jiang H., Yang K., Wang S.Y., Wang R., Li S., Lei P., Xu H., Qiu Y.B. (2021). Whole genome sequencing and annotation of *Naematelia aurantialba* (Basidiomycota, edible-medicinal Fungi). J. Fungi.

[8] Li S.J., Meng G.L., Dong C.H. (2022). Homokaryotic high-quality genome assembly of medicinal fungi *Wolfiporia hoelen* reveals auto-regulation and high-temperature adaption of probable two-speed genome. Int. J. Mol. Sci..

[9] Dong W.G., Wang Z.X., Feng X.L., Zhang R.Q., Shen Q.Y., Du S.T., Gao J.M., Qi J.Z. (2022). Chromosome-level genome sequences, comparative genomic analyses, and secondary-metabolite biosynthesis evaluation of the medicinal edible mushroom *Laetiporus sulphureus*. Microbiol. Spectr..

[10] Sonnenberg A.S.M., Sedaghat-Telgerd N., Lavrijssen B., Ohm R.A., Hendrickx P.M., Scholtmeijer K., Baars J.J.P., Peer A.V. (2020). Telomere-to-telomere assembled and centromere annotated genomes of the two main subspecies of the button mushroom Agaricus bisporus reveal especially polymorphic chromosome ends. Sci. Rep..

[11] Zhang Y., Huang C.Y., Gao W. (2019). Research advances on molecular mushroom breeding. J. Fungal Res..

[12] Wieloch W. (2006). Chromosome visualization in filamentous fungi. J. Microbiol. Methods.

[13] Schwartz D.C., Cantor C.R. (1984). Separation of yeast chromosome-sized DNAs by pulsed field gradient gel electrophoresis. Cell.

[14] Millis D., McCluskey K. (1990). Electrophoretic karyotypes of fungi: The new cytology. Mol. Plant-Microbe Interact..

[15] Borbye L., Linde-Laursen I., Christiansen S., Giese H. (1992). The chromosome complement of *Erysiphe graminis* f. sp. *hordei* analysed by light microscopy and field inversion gel electrophoresis. Mycol. Res..

[16] Poma A., Pacioni G., Ranalli R., Miranda M. (1998). Ploidy and chromosomal number in *Tuber aestivum*. FEMS Microbiol. Lett..

[17] Wang Y., Sun S.L., Liu B., Wang H., Deng J., Liao Y.C., Wang Q., Cheng F., Wang X.W., Wu J. (2011). A sequence-based genetic linkage map as a reference for *Brassica rapa* pseudochromosome assembly. BMC Genom..

[18] Ethier S.D., Miura H., Dostie J. (2012). Discovering genome regulation with 3C and 3C-related technologies. Biochim. Biophys. Acta.

[19] Tosh J., Tybulewicz V., Fisher E.M.C. (2022). Mouse models of aneuploidy to understand chromosome disorders. Mamm. Genome.

[20] Zhou J.N., Tan C., Cui C., Ge A.H., Li Z.Y. (2016). Distinct subgenome stabilities in synthesized *Brassica* allohexaploids. Theor. Appl. Genet..

[21] Tsai H.J., Nelliat A. (2019). A double-edged sword: Aneuploidy is a prevalent strategy in fungal adaptation. Genes.

[22] Rieseberg L.H., Willis J.H. (2007). Plant speciation. Science.

[23] Knouse K.A., Wu J., Whittaker C.A., Amon A. (2014). Single cell sequencing reveals low levels of aneuploidy across mammalian tissues. Proc. Natl. Acad. Sci. USA.

[24] Forche A., Solis N.V., Swidergall M., Thomas R., Guyer A., Beach A., Cromie G.A., Le G.T., Lowell E., Pavelka N. (2019). Selection of *Candida albicans* trisomy during oropharyngeal infection results in a commensal-like phenotype. PLoS Genet..

[25] Morard M., Macías L.G., Adam A.C., Lairón-Peris M., Pérez-Torrado R., Toft C., Barrio E. (2019). Aneuploidy and ethanol tolerance in *Saccharomyces cerevisiae*. Front. Genet..

[26] Yang F., Teoh F., Tan A.S.M., Cao Y.B., Pavelka N., Berman J. (2019). Aneuploidy enables cross-adaptation to unrelated drugs. Mol. Biol. Evol..

[27] Sionov E., Chang Y.C., Kwon-Chung K.J. (2013). Azole heteroresistance in *Cryptococcus neoformans*: Emergence of resistant clones with chromosomal disomy in the mouse brain during fluconazole treatment. Antimicrob. Agents Chemother..

[28] Gresham D., Desai M.M., Tucker C.M., Jenq H.T., Pai D.A., Ward A., DeSevo C.G., Botstein D., Dunham M.J. (2008). The repertoire and dynamics of evolutionary adaptations to controlled nutrient-limited environments in yeast. PLoS Genet..

[29] Rancati G., Pavelka N., Fleharty B., Noll A., Trimble R., Walton K., Perera A., Staehling-Hampton K., Seidel C.W., Li R. (2008). Aneuploidy underlies rapid adaptive evolution of yeast cells deprived of a conserved cytokinesis motor. Cell.

[30] Selmecki A., Maruvka Y.E., Richmond P.A., Guillet M., Shoresh N., Sorenson A., De S., Kishony R., Michor F., Dowell R. (2015). Polyploidy can drive rapid adaptation in yeast. Nature.

[31] Deng Y.J., Guo L., Lin L.J., Li Y.F., Zhang J.X., Zhang Y., Yuan B., Ke L.N., Xie B.G., Ming R. (2023). Meiosis in an asymmetric dikaryotic genome of *Tremella fuciformis* Tr01 facilitates new chromosome formation. Genome Biol..

[32] Stalpers J.A., Redhead S.A., May T.W., Rossman A.Y., Crouch J.A., Cubeta M.A., Dai Y.C., Kirschner R., Langer G.J., Larsson K.H. (2021). Competing sexual-asexual generic names in *Agaricomycotina* (*Basidiomycota*) with recommendations for use. IMA Fungus.

[33] Papp V., Dai Y.C. (2022). What is the correct scientific name for “Fuling” medicinal mushroom?. Mycology.

[34] Wu F., Li S.J., Dong C.H., Dai Y.C., Papp V. (2020). The genus *Pachyma* (syn. *Wolfiporia*) reinstated and species clarification of the cultivated medicinal mushroom “Fuling” in China. Front. Microbiol..

[35] Wang K.Q., Fu J., Su W., Fang H., Deng F. (2002). Review of Chinese traditional medicinal fungus: *Wolfiporia cocos*. Res. Inf. Tradit. Chin. Med..

[36] Jo W.S., Hong I.P., Yoo Y.B., Park S.D. Improvement on artificial cultivation technique of *Poria cocos* in Korea. Proceedings of the International Mycological Congress 10th IMC10.

[37] Wu F., Zhou L.W., Yang Z.L., Bau T., Li T.H., Dai Y.C. (2019). Resource diversity of Chinese macrofungi: Edible, medicinal and poisonous species. Fungal Divers..

[38] Wang N.N., Zhang Y., Wang X.P., Huang X.W., Fei Y., Yu Y., Shou D. (2016). Antioxidant property of water-soluble polysaccharides from *Poria cocos* wolf using different extraction methods. Int. J. Biol. Macromol..

[39] Shi C.Y., Ma Q.H., Ren M.Y., Liang D.D., Yu Q.T., Luo J.B. (2017). Antitumor pharmacological mechanism of the oral liquid of *Poria cocos* polysaccharide. J. Ethnopharmacol..

[40] Chen B.S., Wang S.X., Liu G.Q., Bao L., Huang Y., Zhao R.L., Liu H.W. (2020). Anti-inflammatory diterpenes and steroids from peels of the cultivated edible mushroom *Wolfiporia cocos*. Phytochem. Lett..

[41] Pu Y.W., Liu Z.J., Tian H., Bao Y.X. (2019). The immunomodulatory effect of *Poria cocos* polysaccharides is mediated by the Ca^2+^/PKC/p38/NF-κB signaling pathway in macrophages. Int. Immunopharmacol..

[42] Luo H.M., Qian J., Xu Z.C., Liu W.J., Xu L., Li Y., Xu J., Zhang J.H., Xu X.L., Liu C. (2020). The *Wolfiporia cocos* genome and transcriptome shed light on the formation of its edible and medicinal sclerotium. Genom. Proteom. Bioinform..

[43] Cao S., Yang Y., Bi G.Q., Nelson D., Hu S., Makunga N.P., Yu B., Liu X., Li X.H., Hu X.B. (2021). Genomic and transcriptomic insight of giant sclerotium formation of wood-decay fungi. Front. Microbiol..

[44] Kim B., Min B., Han J.G., Park H., Baek S., Jeong S., Choi I.G. (2022). Draft genome sequence of the reference strain of the Korean medicinal mushroom *Wolfiporia cocos* KMCC03342. Mycobiology.

[45] Zhang C., Chen L.F., Chen M.T., Xu Z.Y. (2023). First report on the regulation and function of carbon metabolism during large sclerotia formation in medicinal fungus *Wolfiporia cocos*. Fungal Genet. Biol..

[46] Li S.J., Wang Q., Dong C.H. (2021). Distinguishing homokaryons and heterokaryons in medicinal polypore mushroom *Wolfiporia cocos* (Agaricomycetes) based on cultural and genetic characteristics. Front. Microbiol..

[47] Li S.J., Wang Q., Dong C.H. (2022). Bipolar system of sexual incompatibility and heterothallic life cycle in the basidiomycetes *Pachyma hoelen* Fr. (Fuling). Mycologia.

[48] Li S.J., Dong C.H. (2022). A new type of homokaryotic strain of *Wolfiporia hoelen* with indistinguishable phenotypes from the parent strains. Mycosystema.

[49] Li S.J., Dong C.H. (2023). Mating and crossbreeding systems of *Wolfiporia hoelen*. J. Fungal Res..

[50] Jiang X.Y. (2023). Study on the Function of PC-5P-88292_34 and Its Target Gene *CsMYB4* in Tea Tree Response to Pathogen of Water Chestnut Stem Blight. Master’s Thesis.

[51] Yan S.P. (2022). Evaluation of Germplasm Resources and Physiological and Molecular Basis of *Reaumuria soongorica* in Response to Salt Stress. Ph.D. Thesis.

[52] Wang L. (2020). Molecular Mechanisms of Lead Tolerance and Accumulation in Tartary Buckwheat (*Fagopyrum tataricum*). Ph.D. Thesis.

[53] Wang Q. (2020). Transcriptomics Analysis of *Paeonia lactiflora* in Response to Drought Stress and Functional Study of Related Transcription Factors. Ph.D. Thesis.

[54] Li Y. (2018). Maize-Mediated Interaction between *Fusarium graminearum* and Antagonistic Bacteria. Master’s Thesis.

[55] Lu F.Z. (2023). Mechanism Characterization of the *ZmPP2C26* Gene Alternative Splicing Variants Negatively Regulate Drought Tolerance in Maize. Ph.D. Thesis.

[56] Yin Y.Z. (2023). Study on the Function of ZmJMJ20 in Cadmium Tolerance of Maize. Master’s Thesis.

[57] Zhang S.P. (2023). Comparative Transcriptomics Provide Insights into the Pathogenic Immune Response Mechanism of Leaf Brown Spot in Weeping Forsythia. Master’s Thesis.

[58] Han Y.Q., Wu X.X., Jiang S.M., Wei A.Q., Tian N.N., Wang H. (2024). Exploring LRR-RLK genes involved in resistance of foxtail millet to *Sclerospora graminicola* based on the weighted gene co-expression network analysis. Acta Phytopathol. Sin..

[59] Yu Z.F. (2023). Study on Response Mechanism of ‘Wanfeng’ Almonds to Low Temperature Stress Based on Transcriptional and Metabolic Analysis. Ph.D. Thesis.

[60] Jiang Y.L. (2023). Effects of Cold Stress on Adaptability of Oat. Master’s Thesis.

[61] Pan N.H., Guan L.R., Li H.L., Zhao J., Li C.Y., Wang Y.Y., Xie Y. (2024). Screening of blast resistance-related candidate genes in rice landrace ‘Acuce’. Mol. Plant Breed..

[62] Zuo Y.Y. (2023). Evaluation of Mulberry Anthracnose Resistance and Mining of Resistance Related Genes. Master’s Thesis.

[63] Wang S.C. (2023). Physiological and Molecular Mechanisms of Exogenous Thiamine Alleviating Drought Stress in *Pterocarya stenoptera*. Master’s Thesis.

[64] Zhang W. (2022). Cadmium Resistance and Regulatory Pathway of BpTT2-Overexpressed *Broussonetia papyrifera*. Ph.D. Thesis.

[65] Guo X.L. (2023). Effects of TaGSK3 on Wheat Development and Stress Adaptation. Ph.D. Thesis.

[66] Zhao H.C. (2020). Analysis of Genome and Mycoparasitic Mechanism of the Mycoparasite *Coinithyrium minitans*. Ph.D. Thesis.

[67] Wang B. (2018). Drought Tolerance-Related Association Mapping and Natural Varitions of two *MYB* Genes in Cassava (*Manihot esculenta* Cranz). Ph.D. Thesis.

[68] Liu Y. (2022). Functional Analysis of Apple bHLH137-like Gene in Drought and Salt Stress. Master’s Thesis.

[69] Qasim M.U. (2020). Identification of Resistance Genes and Pathways Involved in Resistance for Sclerotinia Stem Rot in *Brassica napus*. Ph.D. Thesis.

[70] Jiang S.Q. (2020). Meta analysis of Core Response Genes in Maize Induced by Abiotic and Biotic Stress. Master’s Thesis.

[71] Guo Z.H. (2019). Identification of Cold Tolerance Associated Areas and Candidate Genes at the Booting Stage of Rice in Cold Region. Ph.D. Thesis.

[72] Zhou D. (2018). RNA-seq Analysis of Potato Leaves during the Early Stage of *Phytophthora infestans* Infection. Master’s Thesis.

[73] Li H.L. (2017). The Transcriptome Analysis of Apple Resistance to *Diplocarpon mali*. Master’s Thesis.

[74] Dong H.H. (2019). Research of Brazilian Resistance Differences to *Fusarium oxysporum* f.sp. *cubense* Race 1 and Race 4. Ph.D. Thesis.

[75] Wang X. (2023). The Molecular Basis of Salicylic Acid Regulating Alfalfa Response to Low Temperature. Ph.D. Thesis.

[76] Qiu T. (2022). Study on Salt Tolerance and Molecular Regulation Mechanism of Allotriploid *Populus cathayana*. Ph.D. Thesis.

[77] Wei C.M. (2023). Physiological Response and Transcriptome Analysis of *Echeveria* to Low Temperature Stress. Master’s Thesis.

[78] Chen X.J. (2023). The Mechanism of Ascorbic Acid Regulating the Adaptation of Tomato Seedlings to Salt Stress. Ph.D. Thesis.

[79] Li W.Y. (2023). Study on Resistance Response Mechanism of *Sansevieria trifasciata* var. *laurentii* to Indoor Benzene. Master’s Thesis.

[80] Zhu Y.X. (2016). Alleviative Effects and Mechanisms of Silicon on Salt Stress-Induced Damage in Cucumber Seedlings. Ph.D. Thesis.

[81] Jiang L. (2019). Study on Panicle Proteomes of Temperature of Rice in Response to High Nighttime Temperature Stress at Early Milky Stage. Master’s Thesis.

[82] Fan Y.L. (2022). Physiological and Molecular Response Mechanism of Potato Tubers to *Fusarium sulphureum* and the Functional Study of StPALs. Ph.D. Thesis.

[83] Luo W. (2023). Identification and Mechanism Analysis of Soda Tolerance of Sweet Sorghum Germplasm Resources. Master’s Thesis.

[84] Xie Y.J. (2018). Analysis of Differentially Expressed Genes and Construction of Gene Co-Expression Network in Rice under Abiotic Stresses. Master’s Thesis.

[85] Li S.J., Dong C.H. (2023). Protoplast monokaryogenesis and cross of the homokaryotic strains of *Wolfiporia hoelen*. Mycosystema.

[86] Walker B.J., Abeel T., Shea T., Priest M., Abouelliel A., Sakthikumar S., Cuomo C.A., Zeng Q.D., Wortman J., Young S.K. (2014). Pilon: An integrated tool for comprehensive microbial variant detection and genome assembly improvement. PLoS ONE.

[87] Chen C.J., Wu Y., Li J.W., Wang X., Zeng Z.H., Xu J., Liu Y.L., Feng J.T., Chen H., He Y.H. (2023). TBtools-II: A “one for all, all for one” bioinformatics platform for biological big-data mining. Mol. Plant.

[88] Marcais G., Kingsford C. (2011). A fast, lock-free approach for efficient parallel counting of occurrences of k-mers. Bioinformatics.

[89] Ranallo-Benavidez T.R., Jaron K.S., Schatz M.C. (2020). GenomeScope 2.0 and smudgeplot for reference-free profiling of polyploid genomes. Nat. Commun..

[90] Simão F.A., Waterhouse R.M., Ioannidis P., Kriventseva E.V., Zdobnov E.M. (2015). BUSCO: Assessing genome assembly and annotation completeness with single-copy orthologs. Bioinformatics.

[91] Meyne J., Ratliff R.L., Moyzis R.K. (1989). Conservation of the human telomere sequence (TTAGGG)n among vertebrates. Proc. Natl. Acad. Sci. USA.

[92] Wang M., Meng G.L., Yang Y., Wang X.F., Xie R., Dong C.H. (2024). Telomere-to-telomere genome assembly of Tibetan medicinal mushroom *Ganoderma leucocontextum* and the first *Copia* centromeric retrotransposon in macro-fungi genome. J. Fungi.

[93] Li H. (2018). Minimap2: Pairwise alignment for nucleotide sequences. Bioinformatics.

[94] Li H., Durbin R. (2010). Fast and accurate long-read alignment with burrows-wheeler transform. Bioinformatics.

[95] Li H., Handsaker B., Wysoker A., Fennell T., Ruan J., Homer N., Marth G., Abecasis G., Durbin R., 1000 Genome Project Data Processing Subgroup (2009). The sequence alignment/map format and SAMtools. Bioinformatics.

[96] Tarasov A. (2015). Sambamba: Fast processing of NGS alignment formats. Bioinformatics.

