# Supplementary material for: Chromosome-Level Assembly Reveals a Fifteen-Chromosome Aneuploid Genome and Environmental Adaptation Strategy of Chinese Traditional Medical Fungus Wolfiporia hoelen
Source: Int J Mol Sci. 2024 Aug 13;25(16):8786. doi: 10.3390/ijms25168786 (PMC11354754; doi:10.3390/ijms25168786)
Supplement: Supplementary file 1 [file ijms-25-08786-s001.zip › ijms-3061409-supplementary.pdf]

Supplementary material

Manuscript title: Chromosome-level Assembly Reveals a Fifteen-chromosome Aneuploid Genome and Environmental Adaptation Strategy of Chinese Traditional Medical Fungus *Wolfiporia hoelen*

Information

1. Supplementary Figures 1 to 2

2. Supplementary Tables 1 to 2

Supplementary Figures

Supplementary Figure 1.

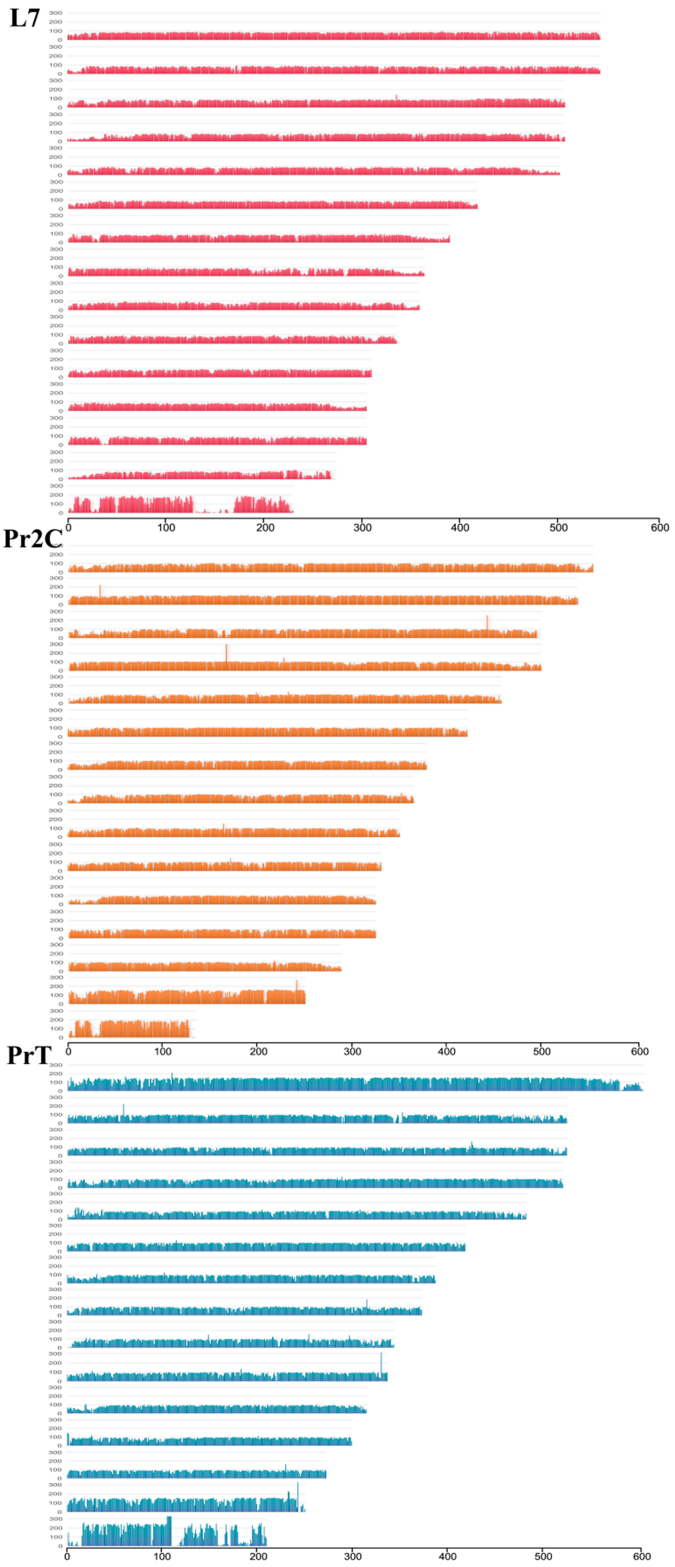

Figure S1. Sequencing depth for 15 chromosomes based on Illumina sequencing reads of *Wolfiporia hoelen*.

Supplementary Figure 2

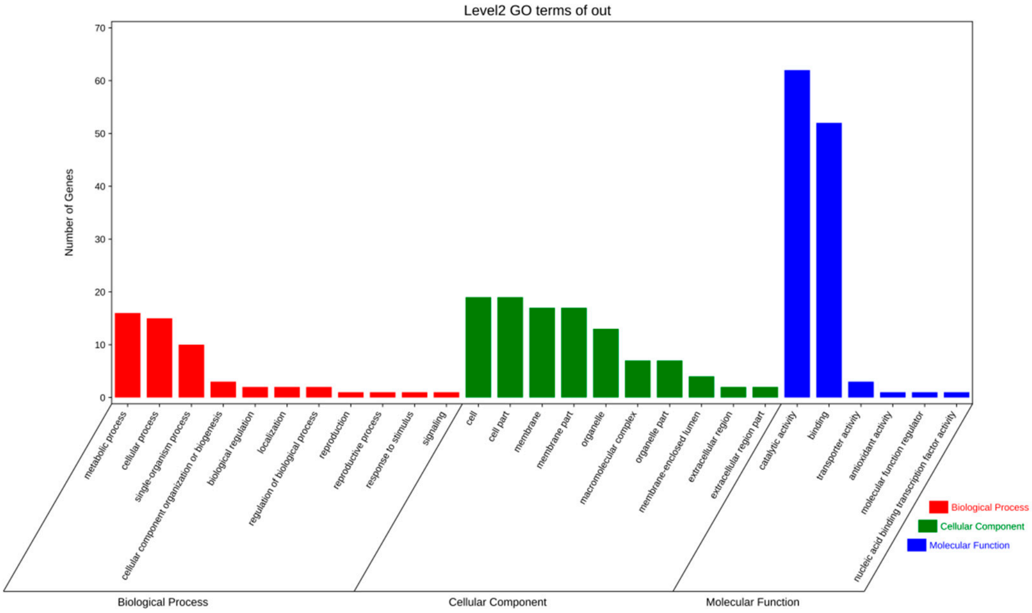

Figure S2. GO enrichment analysis of genes on chromosome 15.

Supplementary Tables

Supplementary Table1

Table S1 Telomere and rDNA sequences distribution for different genomes

**Genome L7**	**end**	**end**	**Genome Pr2C**	**end**	**end**	**Genome PrT**	**end**	**end**	**SS20**	**end**	**end**
Contig1	T	T	Contig1	T	T	Contig1	T	T	Contig1		T
Contig2	T	T	Contig2	T	T	Contig2	T	T	Contig2	T	
Contig3	T	T	Contig3	T		Contig3	T	T	Contig3	T	T
Contig4	T	T	Contig4	T	T	Contig4	T	T	Contig4		T
Contig5	T	T	Contig5	T	T	Contig5	T	T	Contig5	T	T
Contig6	T	T	Contig6	T	T	Contig6	T	T	Contig6	T	T
Contig7	T	T	Contig7	T	T	Contig7	T	T	Contig7	T	T
Contig8	T	T	Contig8	T	T	Contig8	T	T	Contig8	T	T
Contig9	T	T	Contig9	T	T	Contig9		T	Contig9	T	T
Contig10	T	T	Contig10	T	T	Contig10	T	T	Contig10	T	T
Contig11	T	T	Contig11	T	T	Contig11	T	T	Contig11	T	
Contig12	T	T	Contig12	T	T	Contig12		T	Contig12	T	T
Contig13	T	T	Contig13	T	T	Contig13	T	T	Contig13		T
Contig14	T	rDNA	Contig14	T	rDNA	Contig14	T		Contig14	T	rDNA
Contig15	T	T	Contig15	T		Contig15			Contig15		T
Contig16			Contig16	T	T	Contig16	T		Contig16		
Contig17			Contig17			Contig17		T	Contig17	T	
Contig18			Contig18			Contig18			Contig18		T
Contig19			Contig19			Contig19			Contig19		
Contig20			Contig20			Contig20			Contig20		
Contig21			Contig21			Contig21			Contig21		
Contig22			Contig22			Contig22			Contig22		
Contig23			Contig23			Contig23			Contig23	T	
Contig24			Contig24			Contig24			Contig24	T	
Contig25			Contig25			Contig25			Contig25		
Contig26			Contig26			Contig26			Contig26		
Contig27			Contig27			Contig27			Contig27		T
Contig28			Contig28			Contig28			Contig28		

Note: “T” represent telomeres.

Supplementary Table2

Table S2 Basic information of different chromosomes of genome L7

Chromosome ID	Chromosome length	Gene number	Gene density (/kb)	GC content	Repetitive number	Repetitive length	Repetitive ratio
Chr01	5459116	1352	0.25	0.53	2725	1564798	28.66%
Chr02	5430413	822	0.15	0.52	4638	3029191	55.78%
Chr03	5062787	1124	0.22	0.53	3105	1910003	37.73%
Chr04	5023543	869	0.17	0.52	3554	2511411	49.99%
Chr05	4944042	771	0.16	0.52	3797	2784530	56.32%
Chr06	4114369	1038	0.25	0.53	1952	1147487	27.89%
Chr07	3822831	731	0.19	0.52	2652	1712371	44.79%
Chr08	3588497	474	0.13	0.52	3461	2293594	63.92%
Chr09	3516521	557	0.16	0.52	2874	1991087	56.62%
Chr10	3282753	467	0.14	0.51	2841	1764901	53.76%
Chr11	3026396	500	0.17	0.52	2295	1373487	45.38%
Chr12	2963480	436	0.15	0.52	2815	1672734	56.44%
Chr13	2903977	438	0.15	0.51	2724	1553530	53.50%
Chr14	2590401	440	0.17	0.53	2042	1375023	53.08%
Chr15	2153867	234	0.11	0.50	2750	1792939	83.24%
